# The End of the Mitchell-Ward Case

**Published:** 1892-09

**Authors:** 


### THE END OF THE MITCHELL-WARD CASE.

The daily papers have acquainted our readers with the terrible details of the killing of Freda Ward by Alice Mitchell in Memphis, in January last. The trial was conducted last month and Alice was acquitted on the plea of insanity. The expert testimony presented the very unusual spectacle of being unanimously concurrent as to her insanity. Alice had inherited tendencies to insanity from her mother who had also shown homicidal inclinations, and the story of her relations with Freda Ward suggests at once a case of sexual perversion. A description of this unnatural, disgusting and nasty condition has lately passed into literature in *Mademoiselle Giraud, My Wife*, by (it is needless to add) an author of the French school.

The last issue of the *Memphis Medical Monthly* contains a full report of the testimony adduced. The experts who reported were: Dr. F. L. Sim, Professor of Medicine in the Memphis Medical College, of Memphis; Dr. John H. Callendar, Medical Superintendent of the Central Hospital for the Insane, Tennessee, and Professor of Diseases of the Brain and Nervous System in the University of Nashville and Vanderbilt University; Dr. Michael Campbell, Medical Superintendent of the Eastern Asylum for the Insane at Knoxville; (by deposition) Dr. Frank Ingram, Assistant Superintendent of the New York City Lunatic Asylum; (by deposition) Dr. William A. Hammond, Washington City; and others.

We quote from the testimony of Dr. Callendar, with which the other experts entirely agreed: "I believe the immediate act to have been the result of an attack of hysterical mania—the sudden mood of an insane condition of mind which had existed for a considerable time, varying at different periods in its degree and form, as constitutional insanity frequently does. It was my impression that she did not know the nature of a criminal act of the kind she committed, nor the import of the consequences of the coming trial. If she has any idea of the subject it is a vague one, and she is entirely indifferent about it. I rate her capacity and general intelligence quite low. I am of the opinion that Alice Mitchell, on the day I examined her, was of unsound and feeble mind, and that she is so constitutionally, and most probably incurably so, and that under whatever conditions she may be placed, close observation will show that her disorder is, in the main, progressive, and that her intellect will become more feeble. I hold her to have been maniacally insane on the 25th of January last when she slew Freda Ward, and that when I examined her her mind was weak and insane and still the subject of the delusion under which that maniacal fury was engendered."

The poor girl, whose existence was made miserable by a strange and unnatural jealousy, seems to have been the victim of an uncontrollable temptation, or an "emotional morbid impulse," or an "imperative conception." Dominated by this idea she slew her friend.

When last heard from Alice was coolly playing a French harp in the asylum at Bolivar.

